# Inequalities in Access to and Outcomes of Cardiac Surgery Among Patients with Mental Health Disorders

**DOI:** 10.3390/medsci14020277

**Published:** 2026-05-29

**Authors:** Vasileios Leivaditis, Sofoklis Mitsos, Francesk Mulita, Andreas Maniatopoulos, Nikolaos G. Baikoussis, Ejona Shaska, Chrysa Andrikopoulou, Elias Liolis, Theodora Skoura, Andreas Antzoulas, Ioannis Boucharas, Anastasios Sepetis, Periklis Tomos, Manfred Dahm

**Affiliations:** 1 Department of Cardiothoracic and Vascular Surgery, Westpfalz Klinikum, 67655 Kaiserslautern, Germany; vnleivaditis@gmail.com (V.L.); mdahm@westpfalz-klinikum.de (M.D.); 2 Department of Thoracic Surgery, Attikon General Hospital, National and Kapodistrian University of Athens, 12462 Athens, Greece; sophocmit@yahoo.gr (S.M.); periklistomos@hotmail.com (P.T.); 3 Department of General Surgery, General Hospital of Eastern Achaia—Unit of Aigio, 25100 Aigio, Greece; chrysa661@gmail.com (C.A.); ioannisboucharas@gmail.com (I.B.); 4 Department of Electrical and Computer Engineering, Democritus University of Thrace, 69100 Xanthi, Greece; amaniatopoulos@gmail.com; 5 Department of Cardiac Surgery, Ippokrateio General Hospital of Athens, 11527 Athens, Greece; nikolaos.baikoussis@gmail.com; 6 Department of Psychiatry, “Ali Mihali” Psychiatric Hospital, 9401 Vlora, Albania; zilja.jona@yahoo.it; 7 Department of Oncology, General University Hospital of Patras, 26504 Patras, Greece; lioliselias@yahoo.gr; 8 Medical School, National and Kapodistrian University of Athens (NKUA), Aretaeion Hospital, 11528 Athens, Greece; tskoura@yahoo.com; 9 Department of Surgery, General University Hospital of Patras, 26504 Patras, Greece; up1120106@upatras.gr; 10 Postgraduate Health and Social Care Management Program, Department of Business Administration, University of West Attica, 12243 Athens, Greece; tsepet@uniwa.gr

**Keywords:** cardiac surgery, mental health disorders, health inequalities, severe mental illness, postoperative outcomes

## Abstract

Background: Cardiovascular disease remains the leading global cause of morbidity and mortality. Mental health disorders are common comorbidities that significantly influence how patients access and navigate specialist care. Increasingly, mental illness is recognized not merely as a comorbidity but as a potential driver of inequities in cardiovascular care, affecting diagnosis, referral, procedural management, and long-term secondary prevention. These concerns are particularly relevant in cardiac surgery, where care pathways are complex and resource-intensive. Aims and Objectives: This narrative review examines recent evidence on inequalities in access to cardiac surgery and postoperative outcomes among patients with mental health disorders. Particular emphasis is placed on severe mental illness, mood disorders, anxiety-related conditions, and mixed psychiatric cohorts. Materials and Methods: A structured narrative review approach was employed. PubMed and ScienceDirect were systematically searched for peer-reviewed studies published between 2020 and 2025, including cohort studies, registry analyses, systematic reviews, and meta-analyses. The evidence was synthesized thematically, focusing on access to care, perioperative management, clinical outcomes, underlying mechanisms, ethical considerations, policy implications, and future research directions. Results: Evidence suggests that patients with mental health disorders are more likely to undergo cardiac surgery via emergency pathways, experience longer hospital stays, and have higher rates of readmission. Individuals with severe mental illness are less likely to receive invasive coronary procedures compared to the general population and exhibit higher short- and long-term mortality following acute coronary syndromes. Among psychiatric subgroups, psychosis-spectrum disorders appear to be associated with the greatest excess risk of morbidity, mortality, and adverse long-term surgical outcomes. Conclusions: Patients with mental health disorders face inequities across the entire surgical pathway, including preoperative, perioperative, and postoperative phases. Key contributing factors include stigma, diagnostic overshadowing, fragmented healthcare systems, socioeconomic disadvantage, and insufficiently developed models of integrated care. Addressing these disparities requires redesigned referral pathways, strengthened multidisciplinary collaboration (including cardiology, cardiac surgery, psychiatry, and primary care), and a shift toward interventional research aimed at reducing inequities rather than solely documenting them.

## 1. Introduction

### 1.1. Background

The intersection between mental disorders and cardiovascular disease is clinically significant and characterized by bidirectional, mutually reinforcing effects. Recent evidence indicates that individuals with severe mental illness—including schizophrenia and bipolar disorder—have a reduced life expectancy of approximately 15–20 years compared with the general population, with cardiovascular disease accounting for the majority of this excess mortality [1,2].

Findings from recent meta-analyses and cohort studies further demonstrate that individuals with schizophrenia, bipolar disorder, and major depressive disorder experience myocardial infarction at a younger age, exhibit higher rates of cardiovascular mortality, and are less likely to receive invasive cardiovascular treatments than those without such conditions [1,2,3,4].

This intersection is particularly relevant in the context of cardiac surgery. Surgical interventions—such as coronary artery bypass grafting, valve procedures, major open cardiac operations, endovascular interventions, and device-related therapies—represent the culmination of a complex and multi-stage care pathway. This pathway includes symptom recognition, timely diagnosis, specialist referral, preoperative optimization, appropriate procedural selection, and coordinated postoperative follow-up [5,6].

Deficiencies at any stage of this continuum may accumulate and manifest as delayed presentation, an increased likelihood of urgent or emergency surgery, prolonged hospitalization, an elevated complication risk, and suboptimal recovery outcomes [4,5,6].

In the context of mental disorders, these challenges are amplified for several reasons. First, such conditions are associated with a higher prevalence of modifiable cardiovascular risk factors, including smoking, physical inactivity, obesity, diabetes, and suboptimal adherence to long-term treatment regimens [1,2,7].

Second, patients with severe mental illness are less likely to receive invasive coronary procedures and secondary prevention following acute coronary syndromes, reflecting disparities in access to equitable physical healthcare [3,4].

Third, psychiatric symptoms themselves constitute an additional barrier. Depression, anxiety, psychosis, cognitive impairment, and substance-related disorders may impair communication, complicate informed consent, affect symptom reporting, and hinder perioperative medication management and postoperative recovery [2,5,8].

### 1.2. Rationale

A focused examination of cardiac surgery is warranted, as surgical care represents a particularly demanding “stress test” of equity within healthcare systems. For a patient to receive appropriate operative treatment, the system must accurately identify cardiac pathology, equitably assess procedural candidacy, avoid discriminatory gatekeeping, and ensure comprehensive preoperative evaluation, alongside high-quality postoperative care both in hospital and in the community [6,9]. Individuals with mental health disorders may be vulnerable at any stage of this pathway [5,10].

Access to cardiac surgery is not uniformly distributed across the general population. Recent evidence from England demonstrates that female sex, Black ethnicity, and socioeconomic deprivation are independently associated with reduced access to surgery and poorer postoperative outcomes [6]. When mental illness intersects with these established axes of disadvantage, the result may be compounded inequity [11,12]. This is consistent with broader cardiovascular literature, which shows that individuals with severe mental illness are less likely to undergo revascularization, less likely to receive cardioprotective therapies, and more likely to experience higher mortality following acute coronary syndromes [3,4].

Despite these concerns, the body of research specifically addressing cardiac surgery in patients with mental health disorders remains limited compared with the extensive evidence base in general cardiology and acute coronary syndromes. This gap is important, as it may lead to overreliance on findings from non-surgical settings, while the distinct perioperative needs of psychiatric populations remain insufficiently characterized [5,13]. A narrative review is therefore appropriate, as it allows for the integration of both direct evidence from cardiac surgery and relevant insights from broader cardiovascular care pathways, thereby helping to clarify where and how inequalities arise [3,5,13].

### 1.3. Objectives

The present review aims to provide a comprehensive evaluation of inequalities in access to cardiac surgery and associated outcomes among patients with mental disorders. Specifically, the review explores differences in access to referral and surgical pathways, perioperative management, and postoperative outcomes, as well as the potential mechanisms underlying these disparities. It further considers the ethical and policy implications of inequitable access to surgical care and identifies priorities for future clinical practice and research [2,4,5,14].

Where possible, distinctions are made between psychiatric diagnoses, with particular emphasis on differences between severe mental illness and more common affective and anxiety disorders [5,13].

## 2. Materials and Methods

### 2.1. Study Design

This study was conducted as a structured narrative review aimed at synthesizing and critically interpreting the current evidence on inequalities in access to and outcomes of cardiac surgery among patients with mental health disorders. Unlike formal systematic reviews or meta-analyses, which focus on quantitative pooling, this approach allows for the integration of heterogeneous evidence and the exploration of complex, multifactorial phenomena.

A narrative design was considered most appropriate given the diversity of the available literature, which includes varying psychiatric definitions, heterogeneous cardiac procedural categories, and a combination of direct surgical evidence and indirect findings derived from broader cardiovascular care pathways [3,5]. In addition, substantial variation exists across studies in terms of design, population characteristics, and outcome measures, limiting the feasibility of generating a single, meaningful meta-analytic estimate specific to cardiac surgery.

Instead, emphasis was placed on thematic synthesis, enabling the identification and integration of key patterns related to access to care, perioperative management, clinical outcomes, and underlying mechanisms of inequality [5,13]. This approach facilitates a more comprehensive understanding of disparities across the continuum of cardiac surgical care.

### 2.2. Search Strategy

A structured literature search was conducted to identify relevant peer-reviewed studies published between January 2020 and March 2025. The databases PubMed and ScienceDirect were systematically searched using combinations of keywords and controlled vocabulary related to cardiac surgery and mental health.

Search terms included, but were not limited to, cardiac surgery, coronary artery bypass grafting (CABG), valve surgery, pacemaker implantation, revascularisation, acute coronary syndromes, severe mental illness (SMI), schizophrenia, bipolar disorder, depression, anxiety, health inequalities, referral pathways, postoperative outcomes, readmissions, delirium, stigma, and integrated care [2,5,6].

Search strategies were adapted for each database, and reference lists of key articles were also screened to identify additional relevant studies. Emphasis was placed on contemporary research to ensure relevance to current clinical practice and healthcare systems.

Studies were selected not only for direct evidence related to cardiac surgery but also for their contribution to understanding broader mechanisms of inequality, including psychological, social, and system-level factors, as well as national patterns of access to cardiac surgical care [3,8,10].

### 2.3. Inclusion and Exclusion Approach

Studies were eligible for inclusion if they were peer-reviewed, published in English, and appeared between January 2020 and March 2025. Eligible studies were required to contribute directly to at least one of the following domains: access to cardiac surgery or related invasive cardiac procedures; psychosocial outcomes; perioperative or postoperative outcomes in patients with mental health disorders; mechanisms underlying healthcare disparities; or interventions and policy approaches relevant to integrated cardiovascular and mental healthcare [5,13,14].

Priority was given to high-quality evidence, including multicentre cohort studies, national registry analyses, systematic reviews, meta-analyses, and contemporary narrative reviews with clear methodological frameworks. Studies focusing exclusively on non-cardiovascular conditions or lacking relevance to surgical or perioperative care pathways were excluded.

To ensure alignment with current clinical practice and policy, emphasis was placed on contemporary literature. Earlier primary studies were not used as principal evidentiary sources but were considered selectively where necessary to provide contextual or conceptual background [2,3].

### 2.4. Quality Appraisal and Synthesis Strategy

Given the narrative design of this review, a formal risk-of-bias assessment using standardized tools was not undertaken. However, a qualitative appraisal of methodological rigour was performed to assess the reliability and relevance of the included studies. Greater weight was assigned to studies with robust design and methodology, including national registry analyses, large observational cohorts, and systematic reviews that employed transparent methods and appropriate statistical adjustment for confounding factors [2,6,10].

The synthesis of findings was conducted using a thematic approach. Evidence was organized and integrated across key clinical and conceptual domains, including access to care, preoperative evaluation and optimization, perioperative management, postoperative outcomes, underlying mechanisms of disparity, ethical and policy considerations, and areas requiring further research [3,5]. This approach enabled the identification of consistent patterns and gaps across heterogeneous sources, facilitating a comprehensive interpretation of inequalities within the cardiac surgical pathway. The main characteristics of the studies included in this review, including study design, populations, psychiatric exposures, and key inequality-related findings, are summarized in Table 1 [15,16,17,18,19,20,21,22,23,24,25,26,27,28,29,30,31]. Due to substantial heterogeneity across included studies in terms of design, psychiatric exposures, cardiac procedures, outcome definitions, and statistical reporting methods, quantitative effect estimates were not synthesized in a directly comparable manner within the review tables.

### 2.5. Conceptual Framework

Inequalities in access to and outcomes of cardiac surgery among patients with mental disorders are best understood through a multilevel framework encompassing patient-, provider-, and system-level factors. While system-level influences are often the most visible, these domains interact dynamically and cumulatively across the care pathway [1,2].

Contemporary cardiovascular research in severe mental illness emphasizes that the excess disease burden cannot be attributed solely to individual behaviour. Rather, it arises from the interplay of biological vulnerability, social disadvantage, stigma, fragmented healthcare delivery, and unequal treatment across different stages of care [1,2,15].

At the patient level, psychiatric symptoms may delay help-seeking, impair symptom recognition, and reduce the capacity for effective self-management. These factors can negatively influence adherence to medical therapy both before and after surgery [5,9]. Depression, in particular, involves both behavioural and physiological mechanisms, including reduced physical activity, poorer medication adherence, autonomic dysregulation, increased inflammation, and disturbances of the hypothalamic–pituitary–adrenal axis, all of which may adversely affect cardiovascular outcomes and postoperative recovery [8]. In psychotic disorders, cognitive impairment and social dysfunction may further compromise communication, continuity of care, and engagement with healthcare services [2,13].

At the provider level, diagnostic overshadowing and risk-averse clinical decision-making represent key contributors to inequality. Qualitative evidence suggests that clinicians may misattribute physical symptoms to psychiatric conditions, place less trust in patient-reported symptoms, or prioritize mental health concerns in ways that delay appropriate investigation and treatment [9,10,16]. In cardiovascular care, such dynamics may contribute to lower rates of diagnostic procedures, including coronary angiography, reduced use of revascularization strategies, and delayed referral for surgical evaluation [3,4].

At the system level, fragmentation between psychiatry, primary care, cardiology, and surgical services creates additional barriers to equitable care. Discontinuities in communication and coordination may result in eligible patients failing to progress through the care pathway in a timely manner [5,17]. In addition, the absence of standardized referral pathways and the influence of socioeconomic deprivation may promote patterns of late presentation and emergency-only access to surgical services [6,11].

**Figure 1:**
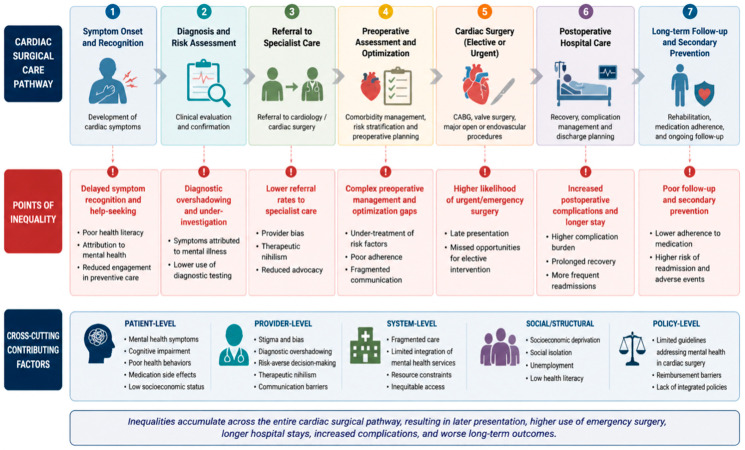
Inequalities across the cardiac surgical care pathway in patients with mental health disorders.

Importantly, these factors do not operate in isolation. Rather, inequalities in cardiac surgery emerge through the cumulative effect of multiple small disadvantages occurring across different stages of care and levels of the healthcare system [5,17]. This multilevel perspective underscores the need for integrated, system-wide approaches to address disparities effectively. These multilevel influences do not act in isolation but accumulate across the entire continuum of care. From initial symptom recognition to long-term postoperative follow-up, patients with mental health disorders may encounter a series of small but consequential barriers that collectively shape access to treatment and clinical outcomes. This cumulative effect is best understood when mapped onto the cardiac surgical care pathway, where inequalities may arise at multiple sequential stages. A schematic overview of this pathway and the key points at which disparities emerge is presented in Figure 1.

## 3. Results

The findings identified in the reviewed literature were synthesized thematically according to major domains of inequality within the cardiac surgical pathway. The following sections summarize evidence related to access to care, perioperative management, postoperative outcomes, and mechanisms contributing to disparities among patients with mental health disorders.

### 3.1. Inequalities in Access to Cardiac Surgery

#### 3.1.1. Referral Patterns and Treatment Selection

An expanding body of evidence indicates that individuals with mental health disorders are less likely to receive timely invasive cardiovascular treatment [3,4]. However, direct evidence specifically addressing referral to cardiac surgery remains limited. In the largest contemporary meta-analysis of patients with severe mental illness following acute coronary syndromes, Chan et al. reported significantly lower odds of receiving any form of revascularization, including coronary artery bypass grafting (CABG), compared with individuals without severe mental illness [3].

These findings are supported by a national cohort study from Scotland, which demonstrated that schizophrenia, bipolar disorder, and major depressive disorder were all associated with reduced rates of coronary revascularization following myocardial infarction. Notably, these disparities persisted over time, suggesting that they have not been eliminated despite advances in cardiovascular care [4,15].

The clinical relevance of these observations lies in the structure of the cardiac surgical pathway. As highlighted by Brooks et al. and Lai et al., CABG is typically performed following a sequence of diagnostic and referral steps that begin earlier in the care continuum [5,6]. Patients with mental health disorders presenting with ischaemic heart disease are less likely to undergo coronary angiography, receive specialist cardiology assessment, or be offered evidence-based revascularization strategies. As a result, they are more likely to progress to surgery through urgent or emergency pathways rather than through planned elective care [3,4].

Indirect evidence from cardiac surgery populations supports this interpretation. In a large South London cohort, patients with a history of mental health service use were significantly more likely to be admitted for cardiac surgery on an emergency basis rather than through elective pathways [5].

Taken together, these findings suggest that inequality in cardiac surgery extends beyond postoperative outcomes to include a distortion in the pathway of access itself [6,14]. Emergency surgical presentation is often the downstream consequence of delayed diagnosis, suboptimal referral, disease progression, or inadequate outpatient management. Consequently, disparities may arise well before patients are formally considered for surgical intervention, reflecting cumulative inequities across earlier stages of care [5,6].

#### 3.1.2. Preoperative Evaluation Barriers

Inequalities may also arise during the preoperative assessment phase, which represents a critical point in determining surgical eligibility and risk. Comprehensive evaluation typically includes both clinical and non-clinical factors, such as treatment adherence, cognitive function, social support, rehabilitation potential, and decision-making capacity [18,19].

Although these assessments are essential, there is a risk that mental illness may be used, implicitly or explicitly, as a proxy for poor surgical candidacy rather than being considered as one of several factors requiring individualized management. This approach may inadvertently disadvantage patients with psychiatric disorders [9,10]. The issue is particularly relevant in conditions such as psychotic disorders, dementia-spectrum disorders, substance use disorders, and severe mood disorders—several of which were represented in the mental health cohort of the South London study [5].

Mild cognitive impairment (MCI), particularly in older adults, may represent an underrecognized contributor to perioperative vulnerability in cardiac surgery populations. MCI is generally defined as measurable cognitive decline that exceeds expected age-related changes while largely preserving functional independence. Importantly, MCI exists on a spectrum distinct from established dementia and should not automatically preclude consideration for major cardiac procedures when clinically indicated. In addition, depressive symptoms and cognitive impairment may overlap clinically, and depression may occasionally mimic or coexist with early neurocognitive disorders, potentially complicating preoperative assessment and decision-making. These considerations further support the need for individualized multidisciplinary evaluation in elderly patients with psychiatric or cognitive comorbidity.

Evidence suggests that patients with mental health disorders may present for surgery with a higher baseline burden of risk. In the South London cohort, these patients were more likely to be older, to be admitted via emergency pathways, and to experience longer index hospital stays, indicating greater preoperative complexity at the time of surgical intervention [5]. Similarly, data from a U.S. institutional cardiac surgery cohort showed that patients with serious mental illness were often younger but had a higher burden of comorbid disease, were more likely to undergo urgent or emergent procedures, and more frequently had a history of prior cardiac surgery, collectively reflecting more complex clinical profiles at presentation [14].

Preoperative management may be further complicated by the use of psychotropic medications. As highlighted by Vu and Smith, abrupt discontinuation of such treatments is not advisable, as it may increase the risk of arrhythmias, bleeding complications, haemodynamic instability, and postoperative neuropsychiatric disturbances [8]. Careful medication reconciliation and coordinated perioperative planning are therefore essential.

Finally, the absence of integrated psychiatric support within perioperative teams may present additional challenges. Limited access to specialist input can hinder optimal risk assessment, perioperative management, and postoperative planning, potentially contributing to less favourable surgical trajectories in this patient population [2,5].

#### 3.1.3. Socioeconomic and System-Level Barriers

Mental health disorders frequently co-occur with socioeconomic disadvantage, including deprivation, unstable housing, unemployment, and limited social support, all of which can significantly influence access to cardiac surgical care [1,2,11]. Evidence from England demonstrates that socioeconomic deprivation independently reduces access to coronary artery bypass grafting (CABG) and valve surgery and is also associated with poorer postoperative outcomes, irrespective of psychiatric diagnosis [6,11].

In this context, individuals with severe mental illness often face a dual burden of medical complexity and structural disadvantage [1,2]. These overlapping vulnerabilities may be further exacerbated by fragmentation within healthcare systems. Cardiac surgical care pathways typically depend on coordinated progression through multiple stages, including diagnostic evaluation, imaging, specialist consultation, preoperative assessment, medication optimization, and postoperative rehabilitation [5,17].

For patients with active psychiatric symptoms or unstable social circumstances, fragmentation across these interfaces may lead to missed appointments, delays in escalation of care, and discontinuities in follow-up [17]. As a result, patients may fail to progress efficiently through the pathway, increasing the likelihood of late presentation and emergency intervention.

Qualitative research in cardiovascular populations further highlights that identifying psychological needs through screening alone is insufficient. Even when referrals are made, patients frequently encounter informational, logistical, and motivational barriers that limit their ability to engage with recommended services [17,20]. These findings underscore the importance of addressing not only clinical factors but also broader social and system-level determinants when seeking to reduce inequities in cardiac surgical care.

#### 3.1.4. Differences by Psychiatric Diagnosis

Not all psychiatric diagnoses confer the same magnitude or pattern of risk in the context of cardiac surgery. Emerging evidence suggests that disparities in access to invasive procedures and adverse postoperative outcomes are most pronounced among patients with psychosis-spectrum disorders [3,13,14]. In a large meta-analysis of patients with severe mental illness following acute coronary syndromes, both schizophrenia and bipolar disorder were associated with reduced rates of revascularization; however, the treatment gap was notably greater in schizophrenia [3].

This gradient is further supported by data from a national Scottish myocardial infarction cohort, in which the likelihood of receiving revascularization was lowest among individuals with schizophrenia, intermediate in those with bipolar disorder, and highest in patients with major depressive disorder [4,15]. These findings indicate that procedural inequities are not uniform across psychiatric conditions but vary according to diagnostic category.

Evidence specific to cardiac surgery, although limited, points in a similar direction. A history of psychosis has been identified as a particularly high-risk factor for adverse postoperative outcomes [14]. In an institutional cardiac surgery cohort, psychosis was associated with significantly increased operative mortality and major morbidity compared with the general surgical population, even after adjustment for baseline risk factors [14].

Mood and anxiety disorders are also clinically relevant but may influence outcomes through different mechanisms. Rather than being associated with the marked procedural disparities observed in psychotic disorders, their impact appears to relate more to factors such as treatment adherence, symptom burden, recovery trajectory, and risk of readmission [5,8]. A structured summary of differences in access to care and outcomes across major psychiatric diagnostic categories is presented in Table 2, highlighting important gradients in risk and patterns of inequality [3,4,5,6,7,8,9,10,11,12,13,14,15,16,17,18,19,20,21,22,23,24,25,26,27,28,29,30,31,32,33,34,35,36,37,38,39].

**Figure 2:**
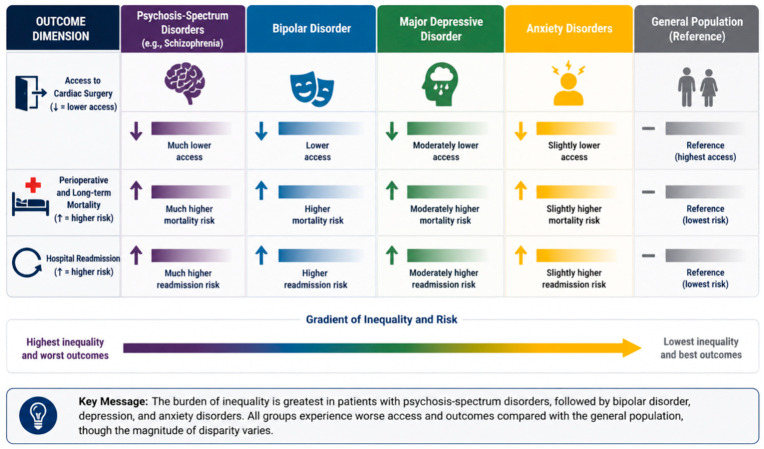
Gradient of inequality in access to and outcomes of cardiac surgery across psychiatric diagnoses. This figure summarizes the relative magnitude of disparities in access to cardiac surgery and key postoperative outcomes across major psychiatric diagnostic groups.

Taken together, the available evidence suggests a clear gradient of inequality across psychiatric diagnoses. Disparities in access to invasive cardiac treatment and adverse postoperative outcomes are most pronounced among patients with psychosis-spectrum disorders, followed by those with bipolar disorder and major depressive disorder, while individuals with anxiety-related conditions generally exhibit more moderate differences. Although all psychiatric groups experience some degree of disadvantage compared with the general population, the magnitude and pattern of inequality vary substantially by diagnosis. A simplified representation of this gradient is shown in Figure 2.

### 3.2. Perioperative Management Differences

#### 3.2.1. Preoperative Optimization

Preoperative optimization in patients with mental health disorders is inherently multidimensional and extends beyond standard cardiovascular risk assessment. It requires integrated evaluation of psychiatric symptoms, medication reconciliation, substance use, sleep patterns, cognitive and decision-making capacity where appropriate, and planning for postoperative support and rehabilitation [2,8,21]. Despite its importance, the literature suggests that such comprehensive, integrated optimization remains insufficiently developed in routine clinical practice [2,5].

Depression has been identified as both a marker of increased perioperative risk and a potentially modifiable factor. Evidence indicates that depressive symptoms are associated with reduced physical activity, poorer medication adherence, lower participation in cardiac rehabilitation, and adverse biological effects, including autonomic dysregulation and inflammatory activation, all of which may contribute to worse surgical outcomes [8]. Failure to identify and address these symptoms prior to surgery may leave patients entering the perioperative period with avoidable vulnerabilities [2,8].

Optimization is particularly challenging in patients with severe mental illness. Factors such as antipsychotic medication use, metabolic comorbidity, high rates of smoking, and social instability may complicate risk reduction and perioperative planning [1,2,7]. As a result, these patients often require more individualized and coordinated approaches to preoperative preparation.

In practice, insufficient integration between psychiatric and surgical care pathways may lead to delays, cancellations, or suboptimal perioperative planning, thereby increasing the risk of postoperative complications and destabilization [22]. These challenges highlight the need for closer collaboration across specialties.

Recent cardiovascular and surgical literature increasingly supports the development of differentiated care pathways tailored to high-risk populations, rather than reliance on uniform management approaches. In this context, multidisciplinary care involving cardiology, cardiac surgery, psychiatry, anesthesia, and primary care is strongly recommended to optimize perioperative outcomes in patients with mental health disorders [1,2,23].

#### 3.2.2. Intraoperative Considerations

Direct evidence regarding intraoperative management differences in patients with mental health disorders undergoing cardiac surgery remains limited, and this absence of data is itself noteworthy [5,13]. While substantial disparities have been documented in preoperative and postoperative care, comparatively little is known about whether intraoperative strategies—such as anesthetic techniques, cardiopulmonary bypass management, haemodynamic control, or monitoring approaches—vary systematically according to psychiatric diagnosis [8,13].

This gap is clinically relevant. As highlighted by Vu and Smith, several factors associated with psychiatric illness may influence intraoperative and immediate postoperative management, including the effects of psychotropic medications, autonomic dysfunction, risk of QT interval prolongation, perioperative agitation, and potential withdrawal syndromes [8]. These considerations suggest that psychiatric comorbidity may introduce additional physiological and pharmacological complexity into the perioperative setting [2,8].

Although direct intraoperative data are scarce, related evidence provides insight into potential mechanisms. Studies focusing on depression in cardiac surgery have identified inflammatory activation, autonomic dysregulation, and adverse behavioural patterns as contributors to poorer outcomes [2,8,24]. Broader reviews of severe mental illness similarly emphasize the cumulative burden of cardiometabolic disease and long-term pharmacotherapy in these populations [1,2].

Taken together, the limited attention to intraoperative factors should not be interpreted as evidence of minimal impact. Rather, it represents a significant gap in the literature. Given the complexity of cardiac surgery and the known physiological and pharmacological challenges associated with mental health disorders, further investigation into intraoperative management is warranted to better understand and address potential sources of disparity [5,13].

#### 3.2.3. Postoperative Care

Postoperative care represents one of the most evident points at which inequalities manifest. Evidence from the South London cohort demonstrates that patients with a history of mental health disorders experience longer index hospital stays and higher rates of 30-day emergency readmission following cardiac surgery, even after adjustment for demographic factors [5].

Similar patterns have been observed across other cardiac procedures, including major open surgery and device implantation, where patients with mental health conditions are more likely to be admitted through emergency pathways and to exhibit less favourable recovery during the initial hospitalization [5]. These findings likely reflect a combination of overlapping factors, including greater baseline clinical complexity, higher rates of urgent surgery, limited discharge support, reduced early recognition of postoperative complications, and barriers to effective engagement with follow-up care [5,20].

In the immediate postoperative period, patients with mental health disorders may face additional challenges, including sleep disturbances, increased susceptibility to agitation and delirium, higher analgesic requirements, and the need for careful coordination of psychopharmacological treatment [8,25,26,27]. These factors can complicate recovery trajectories and increase the risk of adverse events.

Delirium, in particular, warrants special attention. In the general cardiac surgery population, postoperative delirium remains common and is associated with prolonged intensive care unit stays, extended mechanical ventilation, increased complication rates, and higher risks of mortality, as well as long-term cognitive and functional decline [25,26,28]. Patients with mental health disorders may be especially vulnerable, as psychiatric comorbidity frequently coexists with established delirium risk factors, including advanced age, sleep disorders, prior cerebrovascular disease, polypharmacy, and prolonged exposure to intensive care environments.

Despite these concerns, there is a paucity of diagnosis-specific data on postoperative delirium in patients with mental health disorders undergoing cardiac surgery. This represents an important gap in the literature, given the potential impact of delirium on both short- and long-term outcomes in this population [5,25,26].

### 3.3. Postoperative Outcomes

#### 3.3.1. Mortality

The relationship between mental health disorders and postoperative mortality following cardiac surgery is complex and varies across diagnostic groups. While some overall analyses do not demonstrate significantly increased in-hospital mortality when psychiatric conditions are considered collectively, more granular, diagnosis-specific evaluations reveal important differences in risk [5,14]. For example, in the South London cohort, inpatient mortality during the index admission did not differ significantly between patients with and without a documented mental health diagnosis [5].

However, reliance on crude in-hospital mortality alone may underestimate the true extent of disparity. As highlighted by Brooks et al. and Kallio et al., differences in outcomes may become more apparent when longer-term follow-up and diagnostic subgroups are considered [5,13]. Indeed, emerging evidence indicates that certain psychiatric populations experience increased mortality, particularly beyond the immediate perioperative period.

Among these groups, psychosis-spectrum disorders appear to confer the highest risk. In an institutional cardiac surgery cohort, Tyerman et al. reported that a history of psychosis was associated with significantly higher operative mortality and major morbidity compared with the general surgical population, even after adjustment for baseline risk factors [14]. Consistent findings from intensive care studies further suggest that postoperative psychosis is associated with increased morbidity and mortality, underscoring the need for enhanced monitoring and management in this subgroup [29].

Longer-term outcomes also raise concern. A Finnish nationwide case–control study of patients with schizophrenia spectrum disorders undergoing coronary artery bypass grafting (CABG) demonstrated that adverse outcomes persist beyond the index hospitalization, with excess mortality becoming more evident over time [13]. This suggests that early postoperative survival does not necessarily translate into favourable long-term prognosis in this population.

These findings are supported by broader cardiovascular literature, which consistently shows that severe mental illness is associated with higher 30-day and 1-year mortality following acute coronary syndromes, even after adjustment for differences in treatment [3,4,15]. Although such data are not specific to cardiac surgery, they reinforce the broader pattern of increased vulnerability among patients with severe mental illness, even after accessing specialist cardiovascular care [3,4].

Similarly, evidence from general surgical populations indicates that severe mental illness is associated with increased perioperative mortality and major morbidity, although the magnitude of effect varies across studies [3,30]. Taken together, these findings suggest that mortality disparities in cardiac surgery are likely diagnosis-specific, may be underestimated by short-term metrics, and reflect both perioperative and longer-term vulnerabilities.

#### 3.3.2. Morbidity

Patients with mental health disorders appear to be at increased risk of postoperative morbidity following cardiac surgery. Evidence from institutional cohorts indicates that serious mental illness—particularly psychosis—is associated with a higher incidence of adverse postoperative events [14]. In the study by Tyerman et al., this subgroup exhibited the greatest burden of morbidity compared with the general cardiac surgery population [14].

These findings are likely influenced by the clinical context in which such patients present. Both Tyerman et al. and Brooks et al. highlight that individuals with mental health disorders are more frequently subjected to urgent or emergent procedures, have a higher burden of comorbid disease, and often present with more advanced pathology, all of which contribute to increased perioperative complexity and a higher risk of complications [5,14].

Long-term morbidity is also a significant concern. In a nationwide study of patients with schizophrenia spectrum disorders undergoing coronary artery bypass grafting (CABG), outcomes were consistently worse over extended follow-up, suggesting that the burden of morbidity extends beyond the immediate postoperative period [13]. In addition, emerging evidence points to a substantial burden of postoperative neurocognitive and psychiatric complications, including cognitive decline, depressive symptoms, and post-traumatic stress-like manifestations, all of which may delay recovery and impair functional outcomes after cardiac surgery [31].

Broader cardiovascular research further indicates that late complications may be exacerbated by suboptimal secondary prevention. Patients with severe mental illness are less likely to receive or adhere to cardioprotective therapies, which may contribute to ongoing cardiovascular risk despite technically successful surgical intervention [1,2]. Importantly, cohort studies in patients with schizophrenia suggest that appropriate delivery of secondary preventive treatment is associated with improved long-term outcomes, indicating that at least part of the excess morbidity is potentially modifiable [32].

Despite these insights, there remains a lack of detailed, diagnosis-specific data on key postoperative complications—such as stroke, infection, renal injury, reoperation, and wound complications—in psychiatric populations undergoing cardiac surgery [5,13]. This limitation represents a significant gap in the current evidence base and highlights the need for more granular and targeted research.

#### 3.3.3. Length of Stay and Readmissions

Length of hospital stay and readmission rates are among the most consistently reported adverse outcomes in patients with mental health disorders undergoing cardiac surgery. Evidence from the South London cohort demonstrates that a documented mental health diagnosis is associated with prolonged index hospitalization and an increased risk of emergency readmission within 30 days, even after adjustment for demographic and socioeconomic factors [5].

This pattern is consistent with findings from the broader cardiovascular literature. Mental health disorders have been linked to higher 30-day readmission rates following acute myocardial infarction, with particularly strong associations observed in patients with schizophrenia [33]. Although these data are not specific to surgical populations, they suggest that post-discharge vulnerability is a recurring and clinically significant feature of cardiovascular care among individuals with psychiatric conditions [5,33].

The increased burden of prolonged hospitalization and readmission likely reflects the cumulative impact of multiple factors across the care pathway. These include delayed presentation, higher rates of urgent or high-acuity procedures, suboptimal discharge planning, reduced capacity for early recognition of complications, medication-related challenges, and limited access to community-based support [5,20,34].

Beyond their clinical implications, these outcomes contribute to secondary forms of inequity. Prolonged hospital stays and repeated admissions impose additional physical, psychological, and financial burdens on patients and caregivers, while also increasing healthcare resource utilization. Furthermore, repeated disruptions in care may reduce engagement with planned rehabilitation and follow-up, thereby perpetuating a cycle of adverse outcomes [5,20].

#### 3.3.4. Functional Recovery and Quality of Life

Functional recovery and quality of life are clinically important outcomes following cardiac surgery, yet they remain underrepresented in studies involving patients with mental health disorders [35,36]. Existing evidence suggests that recovery trajectories are often influenced not only by surgical success but also by psychological and social factors.

Patients with cardiovascular disease frequently experience preoperative discomfort and psychological distress, which may persist into the postoperative period. Recovery can be prolonged, and ongoing psychological symptoms may hinder participation in rehabilitation, contribute to misinterpretation of physical symptoms, and impair daily functioning after discharge [36].

Emerging evidence indicates that targeted interventions, including psychological support and digital follow-up strategies, may improve depressive symptoms and certain quality-of-life domains after surgery, although the overall quality of evidence remains moderate to low [35,36]. These interventions may be particularly relevant for patients with pre-existing mental health disorders, who are at increased risk of suboptimal recovery.

Importantly, technical success of the surgical procedure does not necessarily translate into meaningful functional recovery. Population-based studies using patient-reported outcome measures show that, while many patients experience improvement, a substantial proportion report persistent limitations in quality of life up to one year after surgery [37]. These findings highlight the distinction between procedural success and patient-centred recovery.

Although many patients are able to achieve adequate levels of mobility, self-care, treatment adherence, and engagement in rehabilitation, several factors may limit recovery potential. These include depression, anxiety, psychosis, cognitive impairment, and adverse social conditions. Despite their relevance, contemporary surgical studies rarely report stratified outcomes based on psychiatric status, nor do they consistently capture functional trajectories or long-term quality-of-life endpoints [35,36,38].

This lack of detailed, patient-centred outcome data represents a significant limitation of the current literature and underscores the need for more comprehensive evaluation of recovery in psychiatric populations undergoing cardiac surgery.

### 3.4. Mechanisms Underlying Disparities

#### 3.4.1. Patient-Level Factors

Patient-level mechanisms underlying disparities in cardiac surgery outcomes encompass both biological and behavioural pathways. Depression, for example, has been linked to inflammatory activation, autonomic dysregulation, and neuroendocrine disturbances, and is also associated with adverse health behaviours, including poor diet, reduced physical activity, lower adherence to medical therapy, and decreased engagement with rehabilitation [8].

Patients with severe mental illness are frequently exposed to additional risk factors, including high rates of smoking, metabolic syndrome, and the cardiometabolic effects of psychotropic medications, as well as challenges in effective self-management [1,2,7]. These factors contribute to an elevated baseline cardiovascular risk and may complicate both preoperative optimization and postoperative recovery.

Delayed presentation represents another important mechanism. Individuals with mental health disorders may underreport symptoms, misinterpret their severity, avoid seeking care, or encounter difficulties navigating healthcare systems, leading to presentation at more advanced stages of disease [5,9]. This pattern is reflected in higher rates of emergency surgical admissions. In the South London cohort, patients with a history of mental health service use were more likely to undergo cardiac surgery via emergency pathways [5]. Similarly, population-level data indicate that individuals with psychiatric disorders are more likely to receive emergency surgical care across multiple specialties, suggesting that delayed presentation and restricted access pathways are widespread rather than context-specific [39].

Postoperative recovery is also influenced by the availability of practical and social support. Effective recovery following cardiac surgery requires coordinated management of medications, adherence to lifestyle modifications, wound care, attendance at follow-up appointments, and engagement in rehabilitation programmes [5,20]. Patients with mental health disorders may face additional barriers in these areas, particularly in the presence of social isolation, unstable housing, or cognitive impairment.

**Figure 3:**
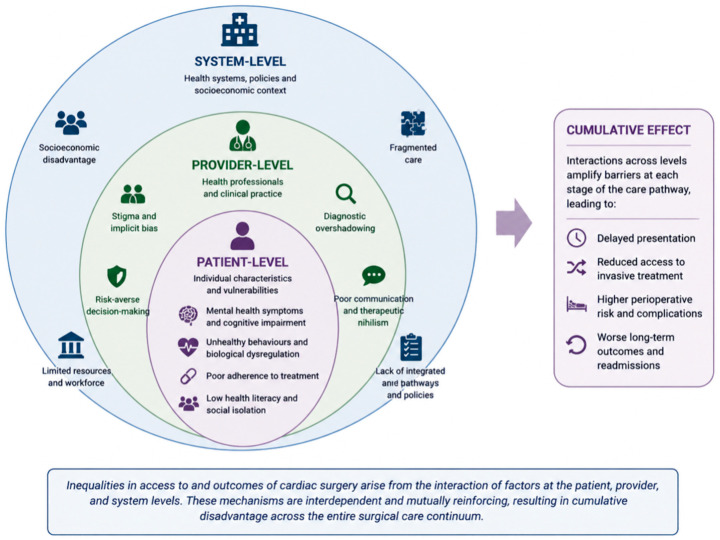
The multilevel mechanisms underlying inequalities in cardiac surgical care. This figure illustrates a simplified conceptual framework of the interacting factors contributing to disparities in access to and outcomes of cardiac surgery among patients with mental health disorders. Inequalities arise across three interconnected levels.

As a result, limited access to adequate support systems may contribute to prolonged hospitalization, increased risk of complications, and higher rates of readmission in this population [5,20,40]. These patient-level factors interact with broader provider- and system-level influences, collectively shaping disparities in cardiac surgical care. These disparities are best understood through a multilevel framework in which patient-, provider-, and system-level factors interact dynamically and reinforce one another. A simplified conceptual representation of these interacting mechanisms is shown in Figure 3.

#### 3.4.2. Provider-Level Factors

Provider-level factors contributing to disparities in cardiac surgical care include stigma, diagnostic overshadowing, and variations in clinical judgement under conditions of uncertainty [16,41]. A growing body of research has documented patterns of suboptimal clinical decision-making in patients with serious mental illness (SMI) and coexisting medical conditions, with recent scoping reviews highlighting the extent and persistence of these issues [10].

Qualitative studies consistently report that patients with mental health disorders often perceive a lack of trust in their accounts, with physical symptoms being attributed to psychiatric conditions and diagnostic or therapeutic interventions being delayed or withheld [9,16]. In cardiovascular care, such dynamics may influence key decision points, including the escalation of symptoms, the use of diagnostic procedures such as coronary angiography, the initiation of revascularization strategies, and ultimately, referral for surgical evaluation [3,4].

Importantly, disparities in care are not always driven by explicit refusal of treatment. Rather, they often arise through more subtle mechanisms, including delays in decision-making, increased scrutiny, and negative assumptions regarding treatment adherence or postoperative cooperation. These factors may contribute to a preference for less invasive or more conservative management strategies, even when more definitive interventions would be clinically appropriate [10,12].

Risk aversion is likely to play a particularly significant role in cardiac surgery, given the high-risk, resource-intensive, and multidisciplinary nature of these procedures [9,10]. Evidence from studies of cognitive processes in surgical teams suggests that decision-making may be influenced by heuristics such as confirmation bias and overconfidence, which can reinforce conservative choices in complex or multimorbid patients [42].

When psychiatric diagnoses are implicitly equated with reduced capacity for postoperative engagement, limited rehabilitation potential, or inadequate social support, clinicians may—consciously or unconsciously—raise the threshold for offering or pursuing surgical intervention [9,10]. Although this mechanism remains underexplored in cardiac surgery specifically, it is well supported by broader research on bias and inequity in healthcare decision-making [10,12].

#### 3.4.3. System-Level Factors

System-level factors play a central role in shaping disparities in access to and outcomes of cardiac surgery among patients with mental health disorders. Evidence from recent reviews consistently highlights inequities across the entire cardiovascular care pathway, ranging from risk factor identification and early screening to acute treatment and long-term secondary prevention [1,2,15].

Fragmentation within healthcare systems represents a key structural barrier. Discontinuities arising from fragmented medical records, limited communication between psychiatric and somatic healthcare services, and insufficient mental health expertise within cardiovascular teams may compromise continuity and safety of care [1,2,5,17]. Such gaps can disrupt patient progression through complex care pathways and increase the likelihood of delayed or suboptimal treatment.

Broader social determinants further interact with these structural challenges. National data from England demonstrate that sex, ethnicity, and socioeconomic deprivation independently influence both access to cardiac surgery and postoperative outcomes [6]. Given that mental health disorders are more prevalent in socioeconomically disadvantaged populations, these factors may converge to produce compounded inequities [2,6,11,12].

This interaction is best understood through the lens of intersectionality. Recent work in cardiovascular health emphasizes that disadvantages related to socioeconomic status, ethnicity, and gender are not simply additive but may combine in multiplicative ways, amplifying their overall impact on health outcomes [43]. In this context, the absence of standardized, integrated care pathways is not a neutral omission but a structural contributor to inequality.

Finally, outcomes may vary considerably depending on contextual factors such as local service availability, institutional culture, clinician attitudes, and the capacity of patients to navigate complex and fragmented healthcare systems during periods of acute illness [5,17,44]. These system-level influences interact with patient- and provider-level factors, reinforcing disparities across the continuum of cardiac surgical care. A summary of the multilevel mechanisms contributing to inequalities in cardiac surgical care is presented in Table 3.

## 4. Ethical and Policy Considerations

At the core of this topic lies the principle of equity in surgical decision-making. Patients with mental health disorders should neither be excluded from potentially beneficial cardiac surgery nor have their psychiatric condition overlooked when it introduces genuine perioperative considerations [2,10]. Achieving this balance requires careful, individualized clinical judgement.

In acute and perioperative settings, psychiatric diagnoses such as depression or psychosis are frequently encountered. An appropriate clinical response lies between two extremes: mental illness should be recognized as clinically relevant and managed with appropriate support, but not treated as a disqualifying characteristic. In particular, assumptions regarding impaired decision-making capacity may distort assessments of informed consent if they are based primarily on diagnosis rather than individual evaluation [9,10,19].

Empirical evidence suggests that decision-making capacity in patients with severe mental illness is influenced more by contextual factors and the availability of support than by diagnosis alone [45]. While many individuals retain full capacity, others may require supported communication or additional time to engage in decision-making. In such cases, concerns about capacity should prompt structured assessment and the use of supportive strategies, rather than automatic restriction of access to definitive treatment options [9,10,19].

The implications of these considerations extend to health policy and service organization. Evidence indicates that patients with mental health disorders undergoing cardiac surgery are more likely to present with higher urgency, experience longer hospital stays, have increased rates of readmission, and receive less access to invasive procedures [2,3,5,6]. These patterns suggest that current models of care are not adequately meeting the needs of this high-risk population.

Consistent findings from broader surgical research reinforce this concern, demonstrating that severe psychiatric illness is associated with higher complication rates, prolonged hospitalization, increased healthcare costs, and more frequent non-elective readmissions [46]. Together, these data point to systemic shortcomings rather than isolated clinical issues.

Addressing these disparities requires coordinated action across cardiology and cardiac surgery services, with integration of mental health expertise into care pathways. In addition, further research is needed to better understand and manage demand for cardiac surgery among vulnerable populations, with a focus on designing equitable and responsive healthcare systems [5].

## 5. Interventions and Strategies to Reduce Inequalities

A central implication of the current evidence is that disparities in cardiac surgical care cannot be addressed through awareness alone; effective interventions must target the entire care pathway rather than focusing solely on individual patients. In this context, integrated care models offer particular promise, as they are specifically designed to reduce fragmentation between mental and physical healthcare systems [17,47].

Systematic reviews indicate that collaborative care approaches can improve access to services, reduce symptom burden, and enhance continuity of care. However, these models have not yet been widely evaluated in patients with mental health disorders undergoing cardiac surgery [17,47,48]. A practical step toward implementation would be the incorporation of structured mental health assessment and support within preoperative pathways for patients with known psychiatric conditions.

Comprehensive preoperative optimization should include assessment by liaison psychiatry or behavioural health specialists, coordinated medication reconciliation involving cardiology and anesthesia teams, evaluation of risk for postoperative delirium or psychiatric relapse, and early discharge planning initiated before surgery [2,5,8]. Such approaches align with broader recommendations for differentiated cardiovascular care in patients with severe mental illness, where standard, uniform pathways may be insufficient [2,5].

Prehabilitation represents another promising strategy. This approach involves preparing patients physically, nutritionally, and psychologically in advance of surgery, with the aim of improving resilience and postoperative outcomes [38]. Evidence from perioperative psychosocial intervention studies in cardiac surgery suggests that structured psychological support may reduce anxiety, lower the risk of delirium, and shorten hospital stay, supporting its potential role in this population [49]. Although the evidence base remains limited, prehabilitation is conceptually well suited to patients with mental health disorders, as it addresses vulnerability before the high-stress perioperative period rather than reacting to complications after they arise [38].

Addressing provider-level factors is equally important. Given the well-documented role of stigma, diagnostic overshadowing, and bias in shaping clinical decisions, targeted training in mental health awareness, supported communication, and equitable decision-making should be incorporated into medical education and professional development, particularly in high-risk specialties such as cardiology, anesthesia, and cardiac surgery [9,10,41]. While such measures are unlikely to eliminate disparities entirely, they may reduce inappropriate gatekeeping and improve interdisciplinary collaboration [10,16].

At the system level, reform should include the development of standardized referral pathways and the routine monitoring of equity indicators. Without systematic measurement—such as tracking delays in referral, severity at presentation, length of stay, and readmission rates—disparities will remain difficult to quantify and address effectively [5,6]. Embedding equity metrics into cardiac surgery programmes is therefore essential to move from descriptive recognition of inequalities toward actionable improvement. A structured overview of proposed interventions across the cardiac surgical care pathway is presented in Table 4.

**Figure 4:**
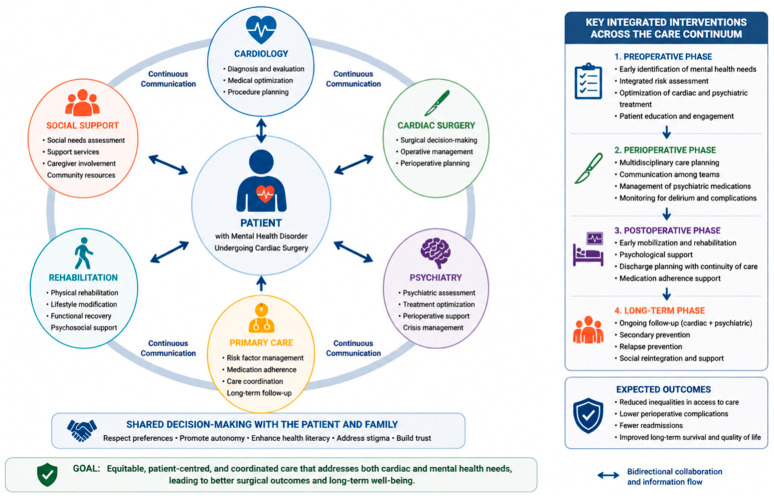
An integrated, multidisciplinary care model for reducing inequalities in cardiac surgery. This figure illustrates a patient-centred, integrated model of care designed to address disparities in access to and outcomes of cardiac surgery among individuals with mental health disorders.

Taken together, the available evidence supports a shift toward integrated, multidisciplinary models of care that address both cardiovascular and mental health needs across the entire surgical pathway. Such models emphasize early identification of psychiatric comorbidity, coordinated perioperative management, and sustained follow-up beyond hospital discharge. Importantly, they rely on close collaboration between cardiology, cardiac surgery, psychiatry, primary care, rehabilitation services, and social support systems. A schematic representation of an integrated care model designed to reduce inequalities in cardiac surgery is presented in Figure 4.

## 6. Discussion

### 6.1. Interpretation of Main Findings

The present review highlights a consistent and clinically meaningful pattern: patients with mental health disorders experience significant inequalities in access to and outcomes of cardiac surgery across the entire continuum of care [3,4,5]. The most robust surgical evidence indicates that these patients are more likely to be admitted through emergency pathways, experience longer hospital stays, and have higher rates of 30-day readmission [5].

When considered alongside the broader cardiovascular literature—particularly studies in acute coronary syndromes demonstrating reduced revascularization rates and increased mortality in severe mental illness—a more comprehensive picture emerges. Psychiatric status appears to influence not only whether patients reach surgical intervention, but also how they access care and what outcomes they experience thereafter [3,4,15].

Importantly, these disparities are not uniform across psychiatric diagnoses. Psychosis-spectrum disorders consistently emerge as the highest risk category, both in cardiac surgery cohorts and in long-term follow-up studies after coronary artery bypass grafting (CABG) [13,14]. This likely reflects the convergence of multiple disadvantage pathways, including social deprivation, healthcare stigma, communication challenges, and an increased burden of cardiometabolic disease [1,2,7,41]. In contrast, mood and anxiety disorders may exert their impact more indirectly, through delayed presentation, reduced adherence, lower engagement with rehabilitation, and an increased likelihood of readmission, rather than through marked differences in operative mortality alone [5,8,50,51].

A key implication of these findings is that postoperative complications represent only one aspect of the problem. Inequities often originate much earlier in the care pathway, including during risk factor management, symptom recognition, referral processes, and access to diagnostic and invasive procedures [2,3,17]. By the time patients present for surgery—often in urgent or critical conditions—these cumulative disadvantages may already be well established.

Addressing these challenges therefore requires a coordinated, system-wide response. Improvements cannot be achieved by surgical teams alone but depend on effective integration across primary care, psychiatry, emergency medicine, cardiology, anesthesia, surgery, and rehabilitation services [2,5,17]. Multidisciplinary collaboration and continuity of care are essential to reducing disparities and improving outcomes.

Several limitations within the existing literature should be acknowledged. The number of studies specifically focused on cardiac surgery in psychiatric populations remains limited, diagnostic definitions are heterogeneous, and long-term patient-centred outcomes are underreported. In addition, it is difficult to fully disentangle the relative contributions of illness severity, comorbidity, and social determinants to observed disparities [5,13].

Despite these limitations, the overall direction of evidence is consistent across different study designs and healthcare settings. This consistency strengthens the validity of the findings and suggests that the true magnitude of inequity may be greater than currently quantified [5,13]. Patients with mental health disorders not only face reduced access to invasive care but also experience more complex clinical pathways, characterized by emergency presentation, prolonged hospitalization, and an increased risk of readmission [5,13,14,33].

These disparities should not be interpreted as the consequence of psychiatric comorbidity alone. Rather, they reflect the interaction of patient-level, provider-level, and system-level mechanisms, including biological vulnerability, stigma, fragmented care, and structural inequities within healthcare systems [1,2,10].

From a clinical and policy perspective, the response must move beyond risk stratification toward an integrated, equity-focused model of care. This includes ensuring fair and timely referral practices, implementing tailored perioperative planning, strengthening discharge and follow-up support, and fostering routine collaboration between cardiovascular and mental health services [2,10,17,38].

The overarching message is clear: equity in cardiac surgery cannot be achieved while mental health remains peripheral to the design and delivery of cardiovascular care [2,6]. The development of mental health–integrated cardiac surgical pathways should therefore be considered a core quality standard, rather than an optional or specialized innovation [2,5,17,52,53,54].

### 6.2. Gaps in the Literature and Future Research

A major limitation of the current evidence base is the relative scarcity of contemporary studies specifically focused on cardiac surgery populations with pre-existing mental health disorders. Much of the available knowledge is derived from broader cardiovascular literature, particularly studies of acute coronary syndromes and invasive cardiology, rather than from cardiac surgery-specific research [3,4]. While this indirect evidence provides valuable insights, it cannot substitute for dedicated investigation within surgical populations [3,5].

A second important gap relates to diagnostic granularity. Many studies group heterogeneous psychiatric conditions under broad categories such as “mental health disorders” or “serious mental illness,” thereby obscuring meaningful differences between diagnoses. Distinctions between schizophrenia-spectrum disorders, bipolar disorder, depression, anxiety disorders, dementia, and substance-related conditions are often not adequately explored [5,14]. Future research should aim to report outcomes by specific diagnostic categories, with particular attention to separating psychotic and non-psychotic disorders. Evidence from broader psychiatric research indicates that such differentiation improves risk stratification and supports more personalized care approaches [5,13,14,50].

A third gap concerns the scope of reported outcomes. Existing studies tend to focus on traditional clinical endpoints such as mortality, length of hospital stay, and readmission rates, while underrepresenting patient-centred outcomes, including quality of life, functional recovery, rehabilitation participation, symptom burden, and psychiatric relapse after surgery [35,36,38]. Given that recovery in this population is strongly influenced by psychosocial factors, this narrow outcome focus represents a significant limitation.

The fourth gap involves the limited understanding of underlying mechanisms. There is a lack of direct evidence linking specific factors—such as clinician bias, referral decision-making processes, medication management, social support structures, and integrated care models—to observed disparities in surgical outcomes [9,10]. Addressing this gap will require the use of mixed-methods approaches and pathway analyses to identify where inequalities arise and which mechanisms are most amenable to intervention [9,10,44].

Finally, there is a notable shortage of high-quality interventional research. Although there is growing support for strategies such as collaborative care, prehabilitation, and integrated perioperative models, few rigorous trials have specifically evaluated these approaches in psychiatric populations undergoing cardiac surgery [17,38]. Future research should therefore move beyond descriptive epidemiology toward implementation studies and pragmatic clinical trials aimed at testing interventions designed to reduce inequities in care [17,38,47].

## 7. Limitations

Several limitations of this review should be acknowledged. First, the study was conducted as a narrative review rather than a formal systematic review or meta-analysis. Although a structured approach to literature selection and synthesis was employed, the absence of a standardized risk-of-bias assessment and quantitative pooling introduces the potential for selection bias and limits the ability to draw definitive causal inferences.

Second, the available evidence specific to cardiac surgery in patients with mental health disorders remains limited. Much of the discussion is therefore informed by broader cardiovascular research, including studies on acute coronary syndromes and invasive cardiology. While this approach provides valuable contextual insight, it may not fully capture the unique aspects of surgical care pathways.

Third, heterogeneity across studies represents an important constraint. Variations in psychiatric definitions, study populations, outcome measures, and healthcare systems limit direct comparability and may affect the generalizability of findings. In particular, the frequent grouping of diverse psychiatric conditions under broad categories such as “mental health disorders” or “serious mental illness” reduces diagnostic specificity.

Fourth, many included studies focus on short-term clinical outcomes, such as mortality, length of stay, and readmissions, while providing limited data on long-term and patient-centred outcomes, including quality of life, functional recovery, and psychiatric relapse. This restricts a more comprehensive understanding of recovery trajectories in this population.

Finally, the observational nature of most available studies limits the ability to disentangle the relative contributions of clinical, social, and system-level factors to observed disparities. Residual confounding related to comorbidity burden, socioeconomic status, and healthcare access is likely to persist across studies.

Despite these limitations, the consistency of findings across multiple sources and settings supports the overall validity of the conclusions and highlights the need for further targeted research in this area.

## 8. Conclusions

Patients with mental health disorders face consistent and clinically significant inequalities in access to and outcomes of cardiac surgery, spanning the entire care pathway from diagnosis to long-term recovery. These disparities are not attributable to a single factor but arise from the interaction of patient-level vulnerabilities, provider-level biases, and system-level fragmentation. Current evidence indicates that affected individuals are more likely to present late, undergo emergency procedures, and experience poorer postoperative trajectories. Addressing these inequities requires a shift from fragmented, reactive care toward integrated, multidisciplinary, and equity-focused models that incorporate mental health into cardiovascular pathways. Future efforts must prioritize targeted interventions, standardized referral processes, and routine monitoring of equity indicators to ensure that mental health status does not remain a determinant of unequal surgical care.

## Figures and Tables

**Table 1:** The characteristics of studies included in the narrative review. This table summarizes key features of the included studies, including study design, setting, population characteristics, psychiatric exposures, and cardiac procedures assessed. It also outlines the primary cardiac outcomes and the main findings related to inequalities in access to care and postoperative outcomes among patients with mental health disorders.

First Author, Year	Country/Setting	Study Design and Data Source	Population and Sample Size	Psychiatric Exposure	Cardiac Procedure/Setting	Key Cardiac Outcomes	Main Inequality-Related Findings
Brooks et al., 2022 [5]	UK, South London secondary mental health services	Retrospective cohort using large mental health and cardiac surgery databases	Adults undergoing cardiac surgery; subset with a history of secondary mental health service use	Any recorded mental health diagnosis (mixed SMI and non-SMI)	Cardiac surgery (CABG, valve, major open procedures, pacemaker)	Index in-hospital mortality, length of stay, 30-day emergency readmission	Mental health service users had more emergency admissions, longer index stay, and higher 30-day emergency readmission; no crude difference in in-hospital mortality.
Lai et al., 2024 [6]	England, National Hospital Episode Statistics	National retrospective registry analysis (2010–2019)	All adults undergoing CABG and valve surgery	Psychiatric diagnosis not primary exposure; mental illness considered within broader axes of deprivation	CABG and valve surgery	Access to surgery, in-hospital survival, postoperative outcomes	Female sex, Black ethnicity, and socioeconomic deprivation were independently associated with reduced access and worse outcomes; mental illness likely intersects with these axes of disadvantage.
Tyerman et al., 2021 [14]	USA, institutional cardiac surgery cohort	Single/multicentre retrospective cohort	Patients undergoing cardiac surgery: a subgroup with serious mental illness	Serious mental illness, with psychosis defined as primary subgroup	Cardiac surgery (including CABG)	Operative mortality, major morbidity	A history of serious mental illness, especially psychosis, predicted significantly higher operative mortality and major morbidity compared with general cardiac surgery population, even after risk adjustment.
Kallio et al., 2022 [13]	Finland, nationwide CABG cohort	Nationwide case–control	Patients with schizophrenia spectrum disorder undergoing CABG matched to controls	Schizophrenia spectrum disorder	CABG	Long-term mortality and major cardiac events	Schizophrenia spectrum disorder associated with impaired long-term outcomes after CABG; excess mortality and morbidity emerged mainly in later follow-up rather than during index admission.
Chan et al., 2022 [3]	International, ACS populations	Systematic review and meta-analysis	Patients with acute coronary syndromes (ACS) with and without severe mental illness	Severe mental illness (schizophrenia, bipolar disorder, major depression)	Invasive coronary management after ACS (not exclusively surgical)	Revascularisation (any and CABG), mortality, cardioprotective pharmacotherapy	Lower odds of receiving any revascularisation and CABG in severe mental illness; higher mortality; treatment gap greatest for schizophrenia.
Fleetwood et al., 2021 [4]	Scotland, national MI cohort	Retrospective cohort using national registries	Patients with myocardial infarction	Severe mental illness (schizophrenia, bipolar disorder, major depression)	Coronary revascularisation after MI	Receipt of revascularisation, mortality	Patients with SMI were less likely to receive revascularisation; post-MI mortality remained higher despite temporal improvements in care.
McBride et al., 2021 [30]	International, multiple surgical specialties	Systematic review and meta-analysis	Patients with serious mental illness undergoing major surgery	Serious mental illness (various diagnoses)	Major surgery, including cardiac procedures	Perioperative mortality and major complications	Serious mental illness associated with higher perioperative mortality and complication rates across surgical fields, including cardiac surgery.
Indja et al., 2017 [31]	Australia, cardiac surgery	Narrative review	Cardiac surgery patients	Psychiatric and neurocognitive morbidity (not always pre-existing)	Cardiac surgery	Neurocognitive and psychiatric sequelae	Highlighted burden of postoperative depression, cognitive decline, and PTSD-like symptoms after cardiac surgery, with implications for patients with pre-existing mental disorders.

Abbreviations: ACS = acute coronary syndrome; CABG = coronary artery bypass grafting; MI = myocardial infarction; PTSD = post-traumatic stress disorder; SMI = severe mental illness; UK = United Kingdom; USA = United States of America.

**Table 2:** A summary of inequalities in access to cardiac surgery and outcomes according to psychiatric diagnosis. This table provides a structured overview of diagnosis-specific patterns of inequality across major psychiatric conditions. It integrates evidence on access to invasive cardiac treatment, perioperative and short-term outcomes, long-term outcomes, and overall patterns of disadvantage, highlighting important differences between psychosis-spectrum disorders, mood disorders, anxiety-related conditions, and mixed psychiatric populations.

Psychiatric Diagnosis	Evidence Base (Examples)	Access to Surgery/Invasive Cardiac Treatment	Perioperative/Short-Term Outcomes	Long-Term Outcomes	Overall Pattern of Inequality
Psychosis/schizophrenia spectrum	Tyerman 2021; Kallio 2022; Chan 2022; Brooks 2022 [3,5,13,14]	Lowest likelihood of receiving revascularisation after ACS; lower CABG rates than non-SMI comparators; more emergency than elective surgical admissions.	Higher operative mortality and major morbidity in cardiac surgery cohorts even after risk adjustment; more emergency procedures; longer index stay; more complex perioperative profiles.	Impaired long-term outcomes after CABG, with excess mortality emerging later; higher ICU morbidity and mortality in postoperative psychosis; sustained cardiovascular risk despite access to specialist care.	Represents the highest-risk group, with pronounced procedural inequity and worse surgical and post-ACS outcomes, likely reflecting combined biological, social, and stigma-related mechanisms.
Bipolar disorder	Chan 2022; Fleetwood 2021 [3,4]; broader SMI cohorts	Lower revascularisation rates after ACS compared to general population, but treatment gap smaller than in schizophrenia; limited direct cardiac surgery data.	Elevated perioperative risk when grouped under SMI but less extreme outcome differentials than psychosis-specific cohorts; data sparse.	Higher cardiovascular mortality than general population; excess risk attenuated but not eliminated by secondary prevention.	Intermediate inequality: clear disadvantage in access and outcomes, but less severe than for psychosis; often merged with other SMI in studies, masking diagnosis-specific nuances.
Major depressive disorder	Chan 2022; Fleetwood 2021; Vu & Smith 2023 [3,4,8]; depression-focused cardiac surgery reviews	Lower receipt of invasive treatment after ACS than non-psychiatric patients, but higher than schizophrenia; limited direct evidence on surgical referral inequity.	Depression is both risk marker and modifiable target; associated with more emergency presentations, more complex perioperative management, higher risk of postoperative delirium, agitation, and depressive relapse.	Linked with poorer functional recovery, reduced participation in rehabilitation, worse quality of life, and increased readmissions; mortality excess smaller than in psychosis but still present.	Inequality expressed mainly through delayed presentation, behavioural risk burden, and impaired recovery rather than dramatically higher operative mortality alone.
Anxiety and related disorders	Vu & Smith 2023 [8]; broader cardiovascular mental-health literature	Less evidence of large treatment gaps in invasive procedures; under-recognized anxiety may still delay help-seeking and affect consent/decision-making.	Contributes to perioperative distress, higher perceived pain, sleep disruption, and delirium vulnerability; may complicate psychotropic management and postoperative monitoring.	Associated with prolonged subjective recovery and reduced quality of life after cardiac surgery; data on hard endpoints limited.	Inequities are subtler and centre on symptom burden, recovery trajectory, and service engagement rather than on large differences in access to surgery.
Mixed/any mental health diagnosis (SMI and non-SMI combined)	Brooks 2022; McBride 2021; Sara 2024; [5,30,39] emergency surgery literature	More likely to undergo emergency surgery; higher urgency classification; across specialties, planned/elective surgery rates are reduced in mental-health service users.	Longer index hospital stay, higher 30-day emergency readmission after cardiac surgery, higher perioperative complications across surgery types.	Higher long-term mortality and morbidity after cardiovascular events; sustained vulnerability due to undertreatment and fragmented secondary prevention.	Confirms a consistent pattern of disadvantage but obscures diagnosis-specific gradients; highlights system-level failure to provide equitable perioperative care.

Abbreviations: ACS = acute coronary syndrome; CABG = coronary artery bypass grafting; ICU = intensive care unit; SMI = severe mental illness.

**Table 3:** The multilevel mechanisms underlying inequalities in cardiac surgical care among patients with mental health disorders. This table summarizes patient-, provider-, and system-level factors contributing to disparities in access to and outcomes of cardiac surgery. It highlights the underlying mechanisms through which these factors operate and their cumulative impact across the surgical care pathway.

Level	Key Factors	Underlying Mechanisms	Impact on Cardiac Surgical Care
**Patient-level**	Depression, psychosis, anxiety, cognitive impairment, substance use disorders; high burden of cardiovascular risk factors (e.g., smoking, obesity, diabetes); social instability	Delayed help-seeking, reduced symptom recognition, impaired self-management, poor adherence to medical therapy, and biological dysregulation (inflammation, autonomic dysfunction, neuroendocrine disturbance)	Late presentation with advanced disease, a higher likelihood of emergency surgery, increased perioperative risk, prolonged recovery, and higher readmission rates
**Provider-level**	Stigma, diagnostic overshadowing, risk-averse clinical decision-making, assumptions regarding adherence or postoperative cooperation	Misattribution of physical symptoms to psychiatric illness, delayed or reduced diagnostic investigation, a lower likelihood of referral for specialist assessment, preference for conservative or less invasive management	Reduced access to angiography, revascularization, and surgical referral; delayed treatment decisions; underutilization of definitive surgical interventions
**System-level**	Fragmentation between mental health and cardiovascular services, lack of integrated care pathways, socioeconomic deprivation, amd disparities related to sex and ethnicity	Poor coordination across care pathways, missed transitions between services, inconsistent follow-up, limited access to specialist care, and structural inequities in healthcare delivery	Delayed or inequitable access to surgery, higher rates of emergency admissions, longer hospital stays, poorer postoperative outcomes, and reduced continuity of care

**Table 4:** Proposed interventions to address inequalities across the cardiac surgical care pathway in patients with mental health disorders. This table outlines key gaps in care at each stage of the cardiac surgical pathway and summarizes targeted interventions aimed at improving equity in access, perioperative management, and long-term outcomes.

Stage of Care	Identified Gaps/Inequalities	Proposed Interventions	Expected Impact
Pre-referral/community care	Delayed presentation, under-recognition of symptoms, poor cardiovascular risk management, limited access to primary care	Integrated primary care models, proactive cardiovascular risk screening in psychiatric populations, improved patient education and outreach	Earlier diagnosis, improved risk factor control, reduced emergency presentations
Referral and diagnostic phase	Reduced access to specialist assessment, angiography, and revascularisation; delayed escalation of care	Standardized referral pathways, equity-focused clinical protocols, decision support tools to reduce bias, improved coordination between primary care and cardiology	More equitable access to diagnostic procedures, timely specialist referral, reduced treatment delays
Preoperative assessment and optimization	Inadequate psychiatric assessment, poor medication management, limited multidisciplinary input, higher baseline risk profiles	Routine involvement of liaison psychiatry, multidisciplinary preoperative clinics, structured medication reconciliation, risk assessment for delirium and psychiatric relapse, individualized optimization plans	Improved perioperative preparedness, reduced complications, better patient selection and risk stratification
Intraoperative management	Limited evidence and lack of tailored approaches for psychiatric populations; potential medication-related and physiological risks	Increased awareness of psychotropic medication effects, individualized anesthetic planning, enhanced monitoring strategies, multidisciplinary perioperative communication	Safer intraoperative management, reduced perioperative instability, improved immediate postoperative outcomes
Postoperative care (in-hospital)	Higher rates of delirium, longer hospital stays, increased complications, fragmented care	Early psychiatric input, structured delirium prevention strategies, coordinated pain and psychopharmacological management, enhanced nursing support	Reduced complications, shorter length of stay, improved early recovery
Discharge and follow-up	Poor discharge planning, limited engagement with follow-up, medication non-adherence, inadequate community support	Structured discharge planning, clear communication with primary care and mental health services, follow-up coordination, patient navigation support	Reduced readmissions, improved continuity of care, better adherence to treatment
Long-term care and secondary prevention	Poor engagement with rehabilitation, undertreatment of cardiovascular risk, fragmented long-term care	Integrated cardiovascular–mental health services, tailored cardiac rehabilitation programmes, digital follow-up interventions, long-term multidisciplinary care models	Improved functional recovery, reduced long-term morbidity and mortality, sustained engagement with care

## Data Availability

No new data were created or analyzed in this study.
